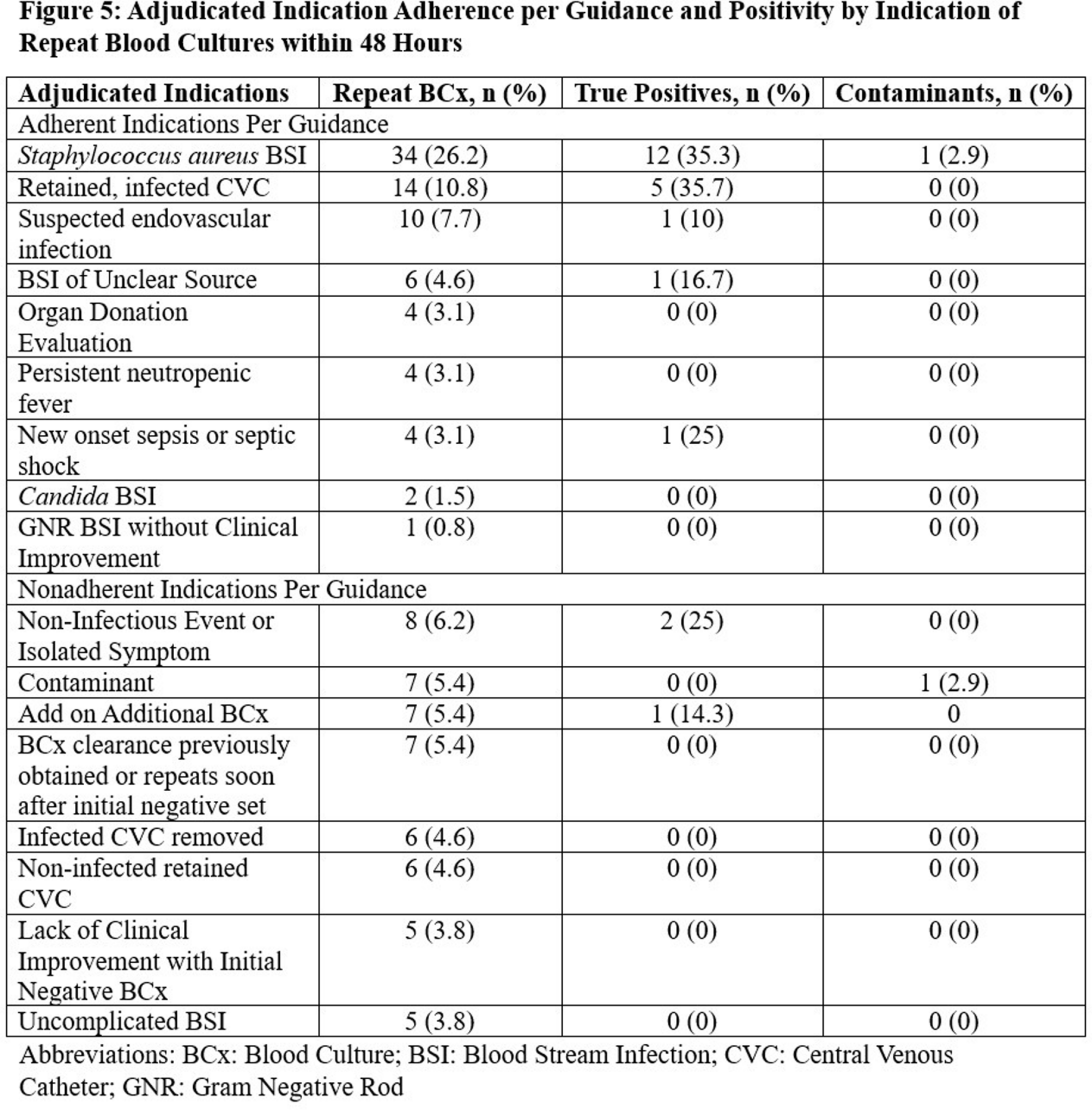# 205 Structure and Leadership of Antibiotic Stewardship Programs in post-acute care: Findings from the APIC MegaSurvey 2025

**DOI:** 10.1017/ash.2026.10592

**Published:** 2026-06-23

**Authors:** Jonathan Ryder, Shawn Freed, Tess Karre, Patty Bruns, Craig Reha, Trevor Van Schooneveld

**Affiliations:** 1 University of Nebraska Medical Center; 2 Enterprise Clinical Applications, Nebraska Medicine; 3 Nebraska Medicine

## Abstract

**Background:** The 2024 blood culture (BCx) bottle shortage highlighted the importance of BCx stewardship. During the shortage period, an electronic medical record alert (Figure 1) was implemented to conserve BCx bottle supply and subsequently maintained after shortage conclusion. An alert fired when repeat BCx were ordered within 48 hours of prior BCx and required written justification of the order to proceed. Sustainable mechanisms to implement BCx stewardship using clinical decision support are needed. This study evaluates whether written justification for bypassing the alert adhered to institutional BCx guidance. **Methods:** A retrospective cohort study of repeat BCx orders where the alert fired and was bypassed in adult patients in first 6 months after implementation (8/12/2024-2/11/2025) was evaluated at a single academic hospital. Primary outcome was the adjudicated adherence to institutional BCx guidance for alert-triggered BCx. Additional outcomes included true positive and contamination rates based on adherence, timing of repeat BCx, and whether repeat blood cultures were obtained <40 hours from initial BCx, serving as a measure of appropriate timing. Falsely triggered alerts were defined as initial BCx orders that were canceled yet triggered the alert or when clinicians added BCx to initial orders within <6 hours. **Results:** Among 162 BCx episodes, 130 were included (exclusions: 7 pediatric, 25 falsely triggered alerts). These 130 BCx episodes consisted of 247 repeat BCx sets in 105 patients. Characteristics of initial and repeat blood culture episodes are in Figure 2. The median time from initial to repeat BCx was 45.6 hours (IQR 35.5-49.3) (Figure 3). Repeat blood cultures were obtained after 40 hours in 67.7% of episodes (Figure 4). Repeat BCx adhered to local guidance for appropriate repeat BCx in 79/130 (60.8%) of episodes with higher yield of true pathogens in adherent indications (20/79, 25.3%) compared to nonadherent indications (3/51, 5.9%) (Figure 5). The indications with highest positivity were retained infected central venous catheters (35.7%) and Staphylococcus aureus bloodstream infection (35.3%). Repeat BCx obtained ≥40 hours had higher adherence compared to those obtained <40 hours (70.5% vs 40.5%). **Conclusion:** Overall adherence to local BCx guidance for repeat BCx via an alert was 60% with higher positivity when ordered for appropriate indications and with appropriate timing. The alert did create falsely triggered alerts, and clinician judgement outside the guidance identified rare cases of persistent bloodstream infection.

**Figure:**
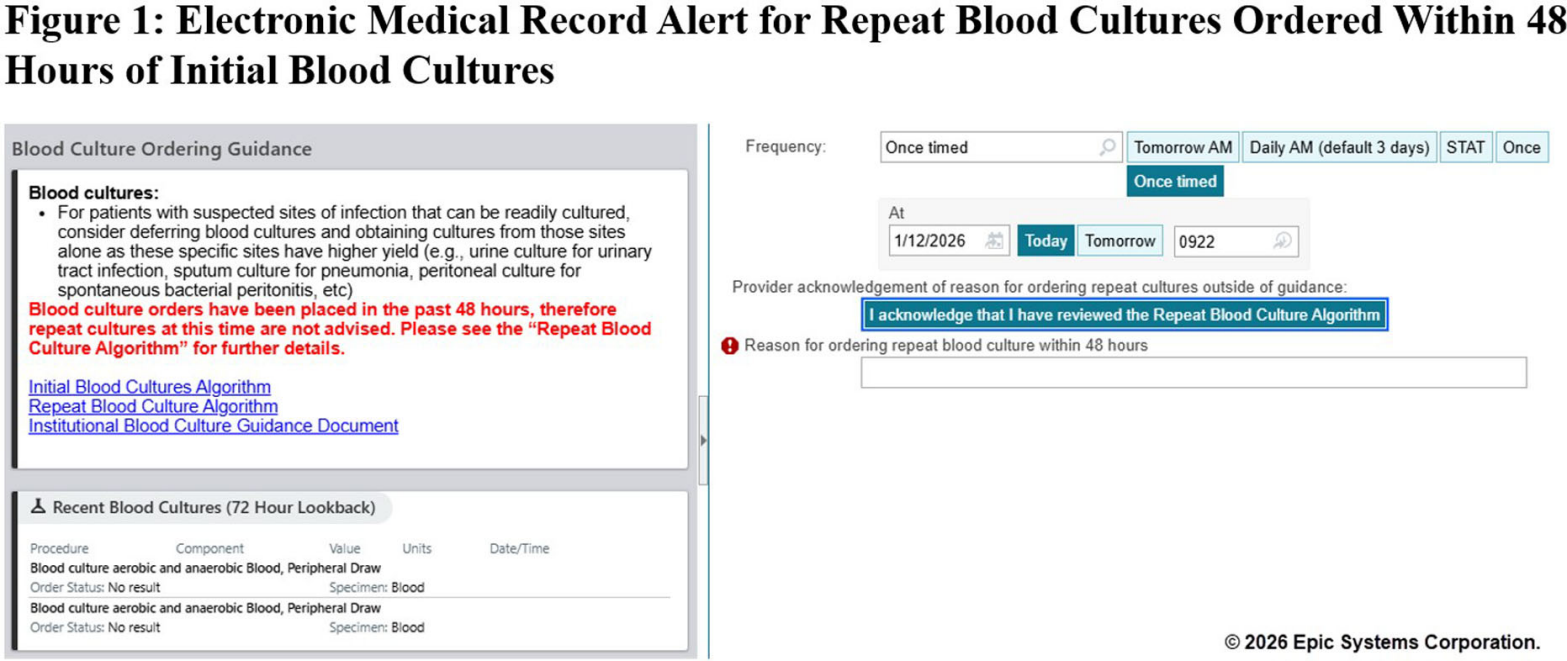

**Figure:**
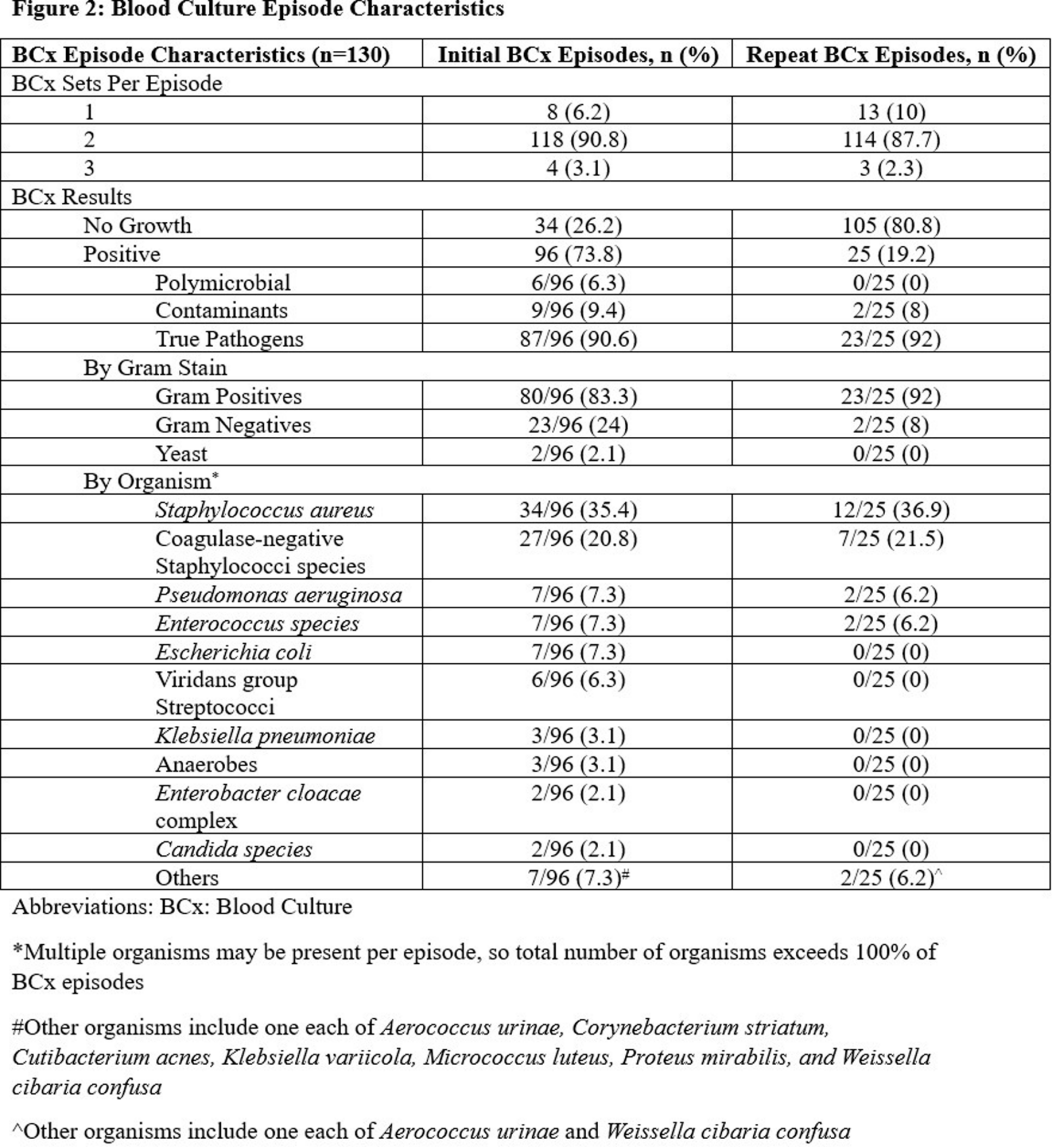

**Figure:**
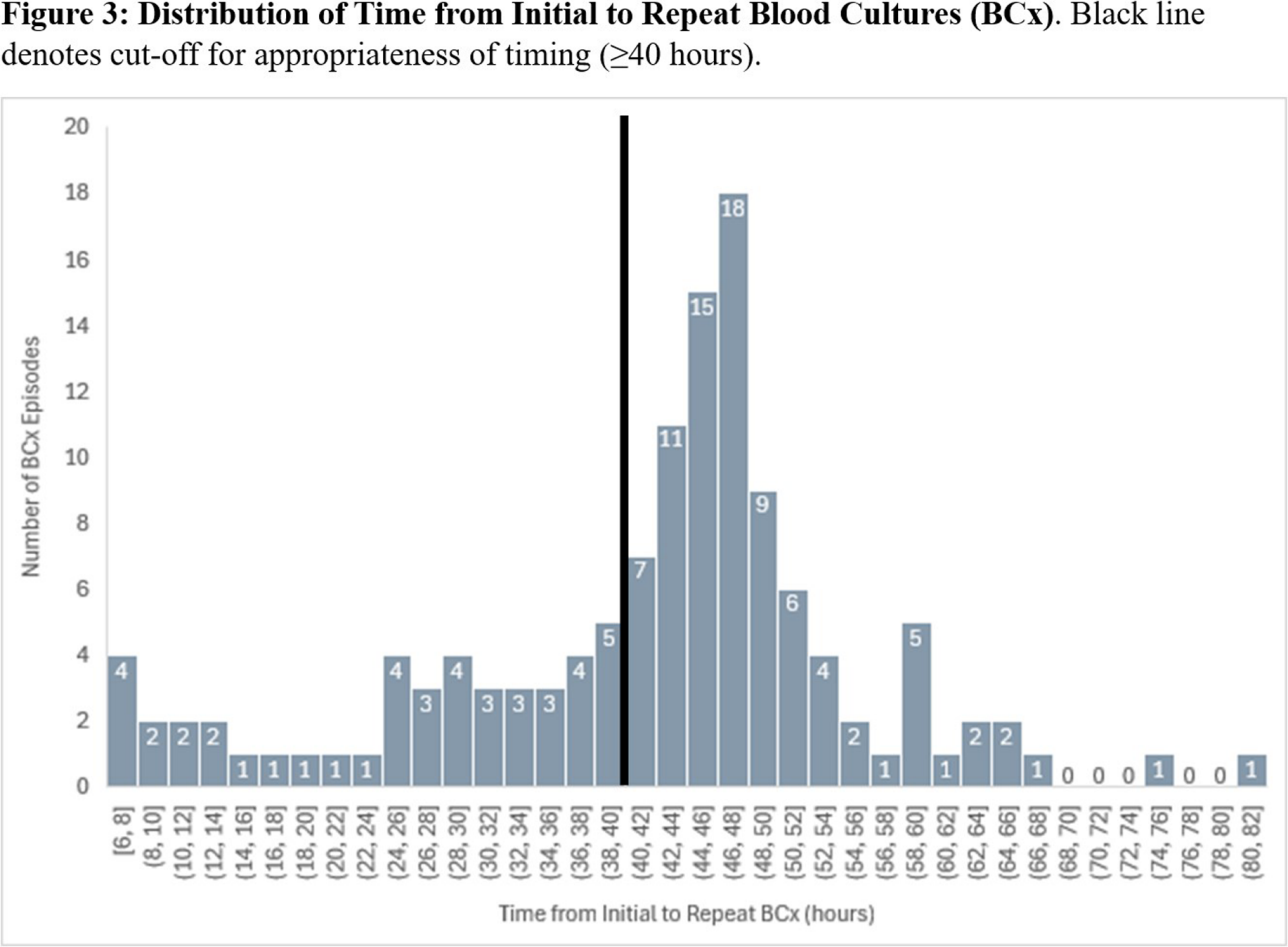

**Figure:**
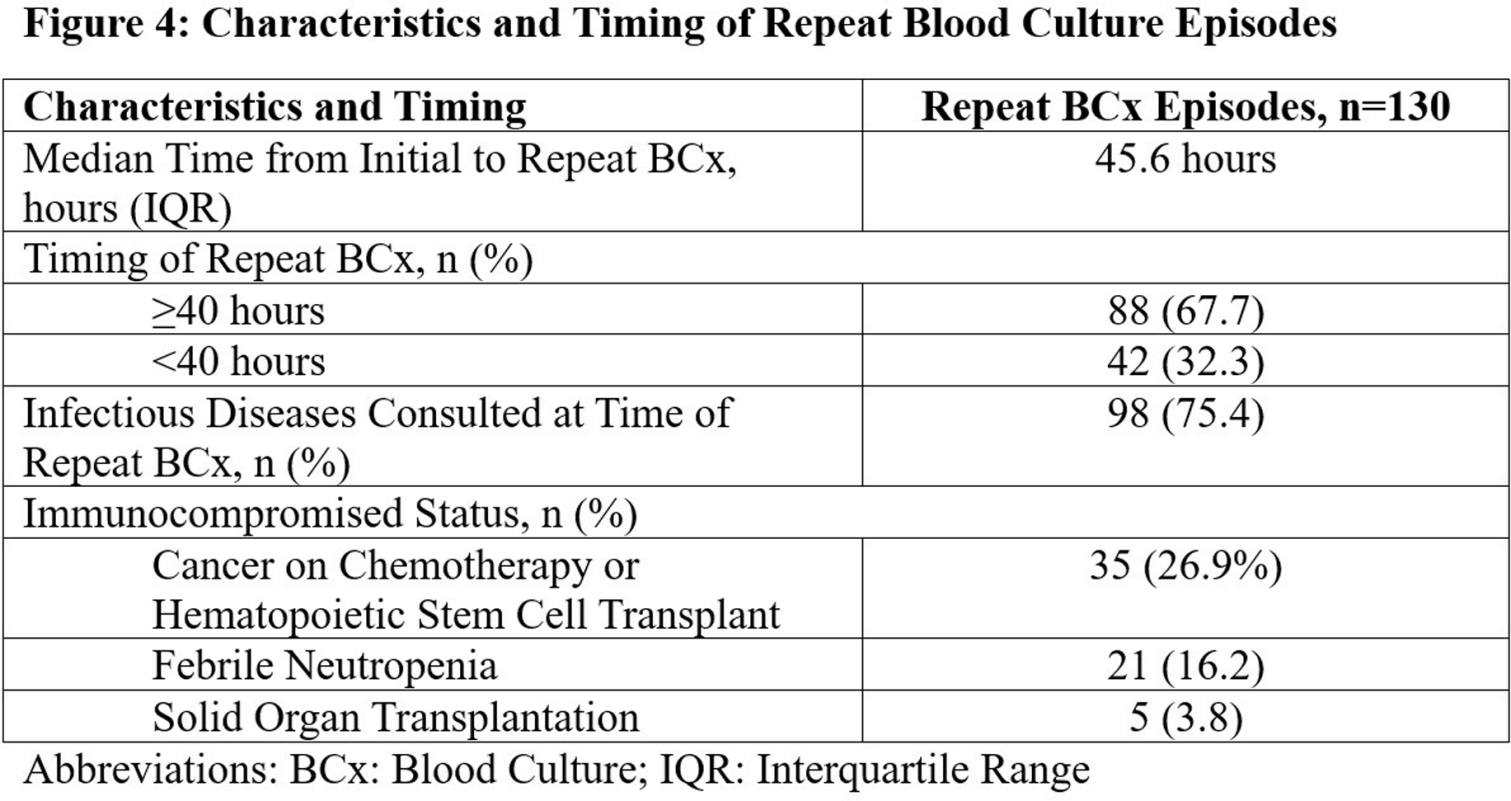

**Figure:**